# Retrospective Evaluation of the Prognosis and Prevalence of Hyperchloremia in Dogs and Cats

**DOI:** 10.1111/vec.70054

**Published:** 2025-11-20

**Authors:** Yu Ueda, Steven E. Epstein, Kate Hopper

**Affiliations:** ^1^ Department of Clinical Sciences, College of Veterinary Medicine North Carolina State University Raleigh North Carolina USA; ^2^ Department of Surgical and Radiological Sciences, School of Veterinary Medicine University of California, Davis Davis California USA

**Keywords:** acid–base balance, acute renal failure, electrolytes, fluid therapy, metabolic acidosis

## Abstract

**Objective:**

To determine the prevalence, case‐fatality rate, and primary disease processes associated with high corrected chloride concentration (hyper[Cl^−^]) in dogs and cats.

**Design:**

Single‐center retrospective study.

**Setting:**

Electrical medical records were reviewed to identify dogs and cats with at least one chloride and sodium concentration measured simultaneously during a 60‐month period.

**Animals:**

A total of 17,120 dogs and 4197 cats presented to a veterinary teaching hospital.

**Interventions:**

None.

**Measurements and Main Results:**

Measured hyper[Cl^−^] was diagnosed in 18.1% (3092/17,120) dogs and 9.4% (396/4197) cats. Corrected hyper[Cl^−^] was diagnosed in 21.1% (3607/17,120) dogs and 9.1% (384/4197) cats. The case‐fatality rates were higher in animals with corrected hyper[Cl^−^] than in those with normal corrected [Cl^−^] (*p* < 0.0001). The case‐fatality rate was higher in dogs with measured hyper[Cl^−^] than in those with corrected hyper[Cl^−^] (*p* = 0.011). Of the dogs and cats with corrected hyper[Cl^−^], a total of 50.9% (1835/3607) dogs and 38.3% (147/384) cats were categorized as prehospital corrected hyper[Cl^−^], whereas a total of 39.5% (1424/3607) dogs and 48.7% (187/384) cats with corrected hyper[Cl^−^] were categorized as hospital‐acquired corrected hyper[Cl^−^]. The case‐fatality rate of dogs and cats with hospital‐acquired corrected hyper[Cl^−^] was higher than that of prehospital corrected hyper[Cl^−^] in dogs (*p* < 0.0001) but not in cats (*p* = 0.9). Various primary disease processes, including neurologic and urologic diseases, were identified in animals with corrected hyper[Cl^−^].

**Conclusions:**

Corrected hyper[Cl^−^] was a common electrolyte abnormality identified in dogs and cats, and it was associated with higher case‐fatality rates than normal corrected [Cl^−^]. Hospital‐acquired corrected hyper[Cl^−^] was less common but was associated with a higher case‐fatality rate than prehospital corrected hyper[Cl^−^] in dogs. Further investigation of corrected hyper[Cl^−^] in association with its morbidity and mortality and the role of therapy to target normal [Cl^−^] is warranted.

**Abbreviations**
AKI: acute kidney injury
CI: confidence interval
CPA: cardiopulmonary arrest
DKA: diabetic ketoacidosis
DM: diabetes mellitus
hyper[Cl^−^]: hyperchloremia
OR: odds ratio

## 1 Introduction

Hyperchloremia (hyper[Cl^−^]) is a common electrolyte abnormality with a high prevalence in critically ill human patients [1]. It is also associated with an increased mortality rate, postoperative complications, readmission rate, and hospital length of stay in ICUs [1, 2, 3, 4]. The development of hyper[Cl^−^] involves mechanisms such as the accumulation of chloride ions and alterations in water balance [1, 2, 5]. The changes in free water balance affect the measured sodium concentration ([Na^+^]) as well as measured chloride concentrations ([Cl^−^]). Therefore, identifying the actual gain of chloride ions, independent of altered water balance, requires the calculation of the corrected ([Cl^−^]) based on the measured [Na^+^] and [Cl^−^] [6, 7].

Corrected hyper[Cl^−^] in clinical patients can arise from various underlying diseases. For instance, it may be caused by chloride retention in cases of renal failure and renal tubular acidosis [8, 9]. Gastrointestinal symptoms, particularly diarrhea, can lead to hyper[Cl^−^] due to the loss of fluid containing bicarbonate and sodium ions with lower chloride ion concentration [10]. In patients with diabetes mellitus (DM) or diabetic ketoacidosis (DKA), corrected hyper[Cl^−^] may result from the retention of chloride ions in place of ketones excreted in the urine [11, 12]. Corrected hyper[Cl^−^] can also develop in patients with hypoadrenocorticism and hypoaldosteronism, where the reduction in measured [Na^+^] is more pronounced than the reduction in measured [Cl^−^] because of aldosterone deficiency [13]. Furthermore, medical treatments involving the administration of chloride‐rich solutions such as 0.9% sodium chloride and potassium chloride, as well as certain diuretics like spironolactone and acetazolamide, have been associated with the development of corrected hyper[Cl^−^] [14, 15].

In veterinary medicine, recent retrospective studies reported a significantly higher case‐fatality rate with corrected hyper[Cl^−^] compared with normal corrected [Cl^−^] in emergency and ICU populations of dogs and cats [16, 17]. Although specific clinical signs directly linked to corrected hyper[Cl^−^] have not been reported in small animals, it is noteworthy that the influence of corrected hyper[Cl^−^] on renal function due to 0.9% sodium chloride administration has been extensively investigated in human patients [18, 19, 20, 21, 22]. These studies have shown that excess chloride ions can negatively affect renal blood flow and glomerular filtration rate. A recent prospective study also reported that approximately 10% of hospitalized dogs developed hyper[Cl^−^] and that hyperchloremic patients were significantly more likely to develop in‐hospital acute kidney injury (AKI). These dogs with hospital‐acquired AKI also had significantly higher maximal corrected [Cl^−^] compared with those without AKI [23].

The aforementioned studies emphasize the significance of corrected hyper[Cl^−^] in both human and veterinary medicine. However, corrected hyper[Cl^−^] in dogs and cats, including the underlying diseases and circumstances contributing to its development, has not been thoroughly characterized. The objective of the current study was to evaluate the epidemiology of corrected hyper[Cl^−^] in a large population of dogs and cats admitted to a tertiary referral hospital, primarily focusing on reporting the prevalence, case‐fatality rate, and underlying disease conditions associated with corrected hyper[Cl^−^].

## 2 Materials and Methods

### 2.1 Case Selection

Medical records from the Veterinary Medical Teaching Hospital at the University of California, Davis, were retrospectively reviewed to identify dogs and cats with recorded plasma or serum chloride concentrations between January 1, 2008, and December 31, 2012. Animals with at least one whole blood or serum [Cl^−^] measured at admission or during hospitalization were further investigated. For the purpose of the current study, the term “blood [Cl^−^] and [Na^+^]” will be used to describe all sample results for simplicity. Animals were admitted through any service at the hospital, and those included in the study could be either systemically healthy or ill, provided that at least one simultaneous blood [Cl^−^] and [Na^+^] measurement was recorded at admission. The raw data containing first sodium and chloride concentrations were extracted from the electronic medical records and transferred to a spreadsheet. Patient signalment, final diagnosis, and outcome (survivor or nonsurvivor, including natural death and euthanasia) were extracted from the medical records. The raw data were visually inspected and manually corrected by removing results from other species or from nonblood samples. Data with missing or erroneous values were also removed from the database. Animals receiving potassium bromide as an anticonvulsant were excluded from the analysis. The curated database included the time and date stamps of each measurement. Only the first admission to the hospital in an animal's lifetime was included if records showed multiple hospital admissions.

### 2.2 Data Collection

Measurement of blood [Cl^−^] and [Na^+^] was performed using a blood gas analyzer^1^ or a serum biochemistry analyzer^2^. Heparinized whole blood samples were immediately used for blood gas analysis, while serum samples were collected in red‐top tubes and promptly submitted to the diagnostic laboratory for serum biochemistry testing. Alternatively, the samples were centrifuged, and the plasma or serum was stored at 4°C until testing, which occurred within 12 h of sample collection.

The reference intervals for measured [Cl^−^] and sodium concentration ([Na^+^]) were previously established based on samples obtained from clinically healthy dogs and cats, considering the respective blood gas or serum biochemical analyzer used.

Corrected chloride concentrations ([Cl^−^]_corrected_) were calculated with the formula [6]:

$${\left[{\text{Cl}}^{-}\right]}_{\mathrm{corrected}}\;=\;{\left[{\text{Cl}}^{-}\right]}_{\mathrm{measured}}\;\times \;\left({\left[{\text{Na}}^{+}\right]}_{\mathrm{normal}}/{\left[{\text{Na}}^{+}\right]}_{\mathrm{measured}}\right)$$

The normal [Na^+^] used for the calculation of corrected [Cl^−^] was determined as the midpoint of the reference interval for measured [Na^+^] for that analyzer and species. Measured [Na^+^] was not corrected for glucose concentration when calculating the corrected [Cl^−^]. Using the formula above, the reference intervals calculated for plasma‐corrected [Cl^−^] were 110–121 mmol/L in dogs and 118–124 mmol/L in cats. These reference intervals were calculated based on blood measured [Cl^−^] from a blood gas analyzer. The reference intervals for corrected [Cl^−^] were 108–116 mmol/L in dogs and 117–126 mmol/L in cats. These reference internals were calculated based on measured [Cl^−^] from a serum biochemical analyzer. Animals with measured or corrected [Cl^−^] concentrations exceeding the reference intervals were categorized as severe (≥10 mmol/L higher than the high end of the reference interval), moderate (7–9 mmol/L higher than the high end of the reference interval), mild (4–6 mmol/L higher than the high end of the reference interval), or borderline (≤3 mmol/L higher than the high end of the reference interval) corrected hyper[Cl^−^]. No definitions for borderline, mild, moderate, or severe chloride disorders were identified in the veterinary literature. Therefore, the categorization used for the severity of hyper[Cl^−^] was chosen based on previous studies in both human and veterinary patients with minor modifications [24, 25].

The onset of corrected hyper[Cl^−^] was categorized as prehospital or hospital‐acquired. Prehospital corrected hyper[Cl^−^] refers to cases where elevated chloride levels were detected on the first blood sample at admission before treatment initiation. Hospital‐acquired corrected hyper[Cl^−^] denotes cases in which the initially obtained corrected [Cl^−^] fell within the reference intervals but subsequently increased during hospitalization. If animals had corrected hyper[Cl^−^] on admission but had received medical treatment by a referring veterinarian within 24 h before referral to our hospital, the timing of the onset of hyper[Cl^−^] was considered unknown. Animals that presented with corrected hyper[Cl^−^] did not require additional samples during the hospitalization. However, animals that presented with normal corrected [Cl^−^] required additional blood tests showing the development of hyper[Cl^−^] during hospitalization to be categorized as hospital‐acquired hyper[Cl^−^].

In cases in which multiple chloride and sodium concentrations were measured during the same hospitalization, the absolute difference between the highest and lowest [Cl^−^] corrected (∆[Cl^−^]_corrected_) was calculated to assess the impact of changes in [Cl^−^] on the outcome, as previously reported in both human and veterinary patients [16, 17, 26]. This analysis involved reviewing all hospital admissions of the animals included in the study and comparing ∆[Cl^−^]_corrected_ values among animals that either survived, died, or were euthanized during their hospital stay.

The medical records of dogs and cats with mild to severe hyper[Cl^−^] were further reviewed to identify the primary disease processes, as determined and written by the primary clinician's final diagnosis in the medical record. These primary disease processes were categorized based on the major organ systems affected, including gastrointestinal, cardiovascular, respiratory, pancreatic, urologic, neurologic, hepatobiliary, oropharyngeal, musculoskeletal, reproductive, and hematologic systems [24]. Specific disease processes such as sepsis, intoxication, neoplasia, hypoadrenocorticism, hyperadrenocorticism, hypothyroidism, hyperthyroidism, DM, DKA, diabetes insipidus, and cardiopulmonary arrest (CPA) were investigated individually due to their multiorgan effects or their specific relevance to abnormal chloride concentrations.

Animals with borderline corrected hyper[Cl^−^] were excluded from this analysis to create a clear distinction between animals with normal corrected [Cl^−^] and those with corrected hyper[Cl^−^]. To determine the prevalence of hyper[Cl^−^] in relation to each specific disease process, the proportion of animals with hyper[Cl^−^] was calculated relative to the total number of animals affected by each primary disease process.

### 2.3 Statistical Analysis

The outcome of animals with or without measured or corrected hyper[Cl^−^] was compared with those with a normal measured or corrected [Cl^−^] using *χ* ^2^ analysis or Fisher exact test where appropriate. Corrected hyper[Cl^−^] was categorized into borderline, mild, moderate, and severe, as previously described. The trend of corrected hyper[Cl^−^] severity and the case‐fatality rate were assessed using the Cochran–Armitage test for trend. As a post hoc analysis, the case‐fatality rates for borderline, mild, moderate, and severe corrected hyper[Cl^−^] were compared with those of animals with normal corrected [Cl^−^] using *χ* ^2^ analysis. The absolute differences in [Cl^−^] corrected (∆[Cl^−^]_corrected_) were compared between survivors and nonsurvivors in animals using either the Student's *t*‐test or the Mann–Whitney *U*‐test, depending on the normality of the data, which was determined by the D'Agostino–Pearson test. The proportion of animals with corrected hyper[Cl^−^] over the total number of animals with each primary disease process was calculated. The association between the primary disease process and the documentation of corrected hyper[Cl^−^] for each primary disease process was analyzed using *χ* ^2^ analysis or Fisher exact test. Bonferroni correction was applied to adjust for multiple comparisons of the primary disease processes. These statistical analyses were conducted using commercially available software^3^. A *p*‐value of <0.05 was considered statistically significant (*p* < 0.002 after Bonferroni correction).

## 3 Results

**FIGURE 1:**
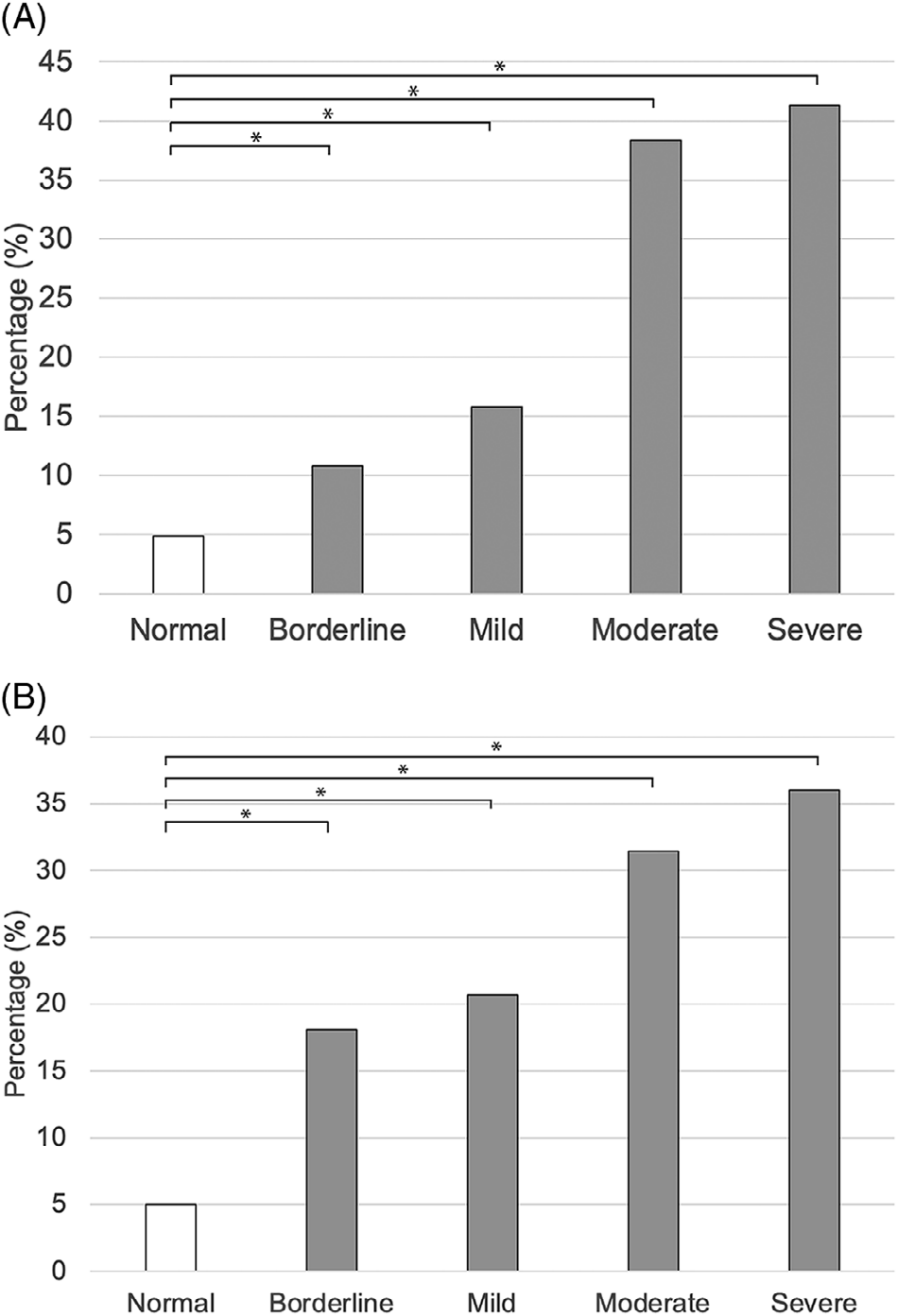
(A) Case‐fatality rates in 14,732 dogs with measured normal chloride concentration and measured hyperchloremia. Significantly higher case‐fatality rates were observed in dogs with measured hyper[Cl^−^] compared with those with normal measured [Cl^−^] (*p* < 0.001). The severity of measured hyper[Cl^−^] showed a linear association with higher case‐fatality rates in dogs (*p* < 0.0001). Dogs with blood or serum measured [Cl^−^] higher than the reference intervals were identified and categorized as severe (≥10 mmol/L higher than the high end of the reference interval), moderate (7–9 mmol/L higher than the high end of the reference interval), mild (4–6 mmol/L higher than the high end of the reference interval), and borderline (<3 mmol/L higher than the high end of the reference interval) measured hyper[Cl^−^]. (B) Case‐fatality rates in 2734 cats with measured normal chloride concentration and measured hyperchloremia. Significantly higher case‐fatality rates were observed in cats with measured hyper[Cl^−^] compared with those with normal measured [Cl^−^] (*p* < 0.001). The severity of measured hyper[Cl^−^] showed a linear association with higher case‐fatality rates in cats (*p* < 0.0001). Cats with blood or serum [Cl^−^]_measured_ higher than the reference intervals were identified and categorized as severe (≥10 mmol/L higher than the high end of the reference interval), moderate (7–9 mmol/L higher than the high end of the reference interval), mild (4–6 mmol/L higher than the high end of the reference interval), or borderline (≤3 mmol/L higher than the high end of the reference interval) measured hyper[Cl^−^].

**FIGURE 2:**
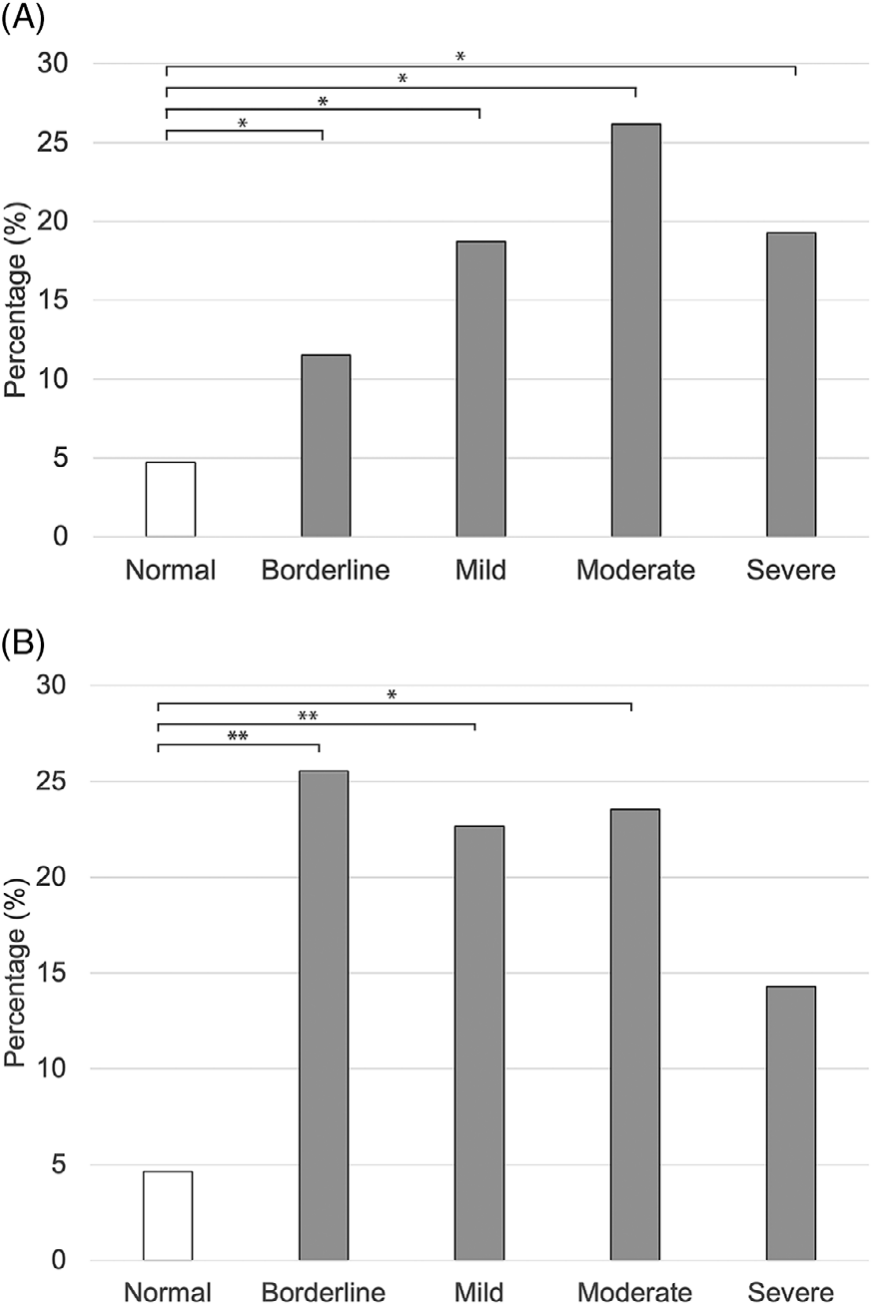
(A) Case‐fatality rate in 14,732 dogs with corrected normal chloride concentration and corrected hyperchloremia. Significantly higher case‐fatality rates were observed in dogs with corrected hyper[Cl^−^] compared with those with normal corrected [Cl^−^] (*p* < 0.001). The severity of corrected hyper[Cl^−^] showed a linear association with higher case‐fatality rates in dogs (*p* < 0.0001). Dogs with blood or serum corrected [Cl^−^] higher than the reference intervals were identified and categorized as severe (≥10 mmol/L higher than the high end of the reference interval), moderate (7–9 mmol/L higher than the high end of the reference interval), mild (4–6 mmol/L higher than the high end of the reference interval), and borderline (<3 mmol/L higher than the high end of the reference interval) corrected hyper[Cl^−^]. (B) Case‐fatality rate in 2734 cats with corrected normal chloride concentration and corrected hyperchloremia. The borderline (*p* < 0.0001), mild (*p* < 0.0001), and moderate corrected hyper[Cl^−^] (*p* = 0.0003) were significantly associated with a higher case‐fatality rate compared with normal corrected [Cl^−^]. However, severe corrected hyper[Cl^−^] was not associated with a higher case‐fatality rate compared with normal corrected [Cl^−^] in cats (*p* = 0.14). The severity of corrected hyper[Cl^−^] did not show a linear association with higher case‐fatality rates in cats (*p* = 0.34). Cats with blood or serum corrected [Cl^−^] higher than the reference intervals were identified and categorized as severe (≥10 mmol/L higher than the high end of the reference interval), moderate (7–9 mmol/L higher than the high end of the reference interval), mild (4–6 mmol/L higher than the high end of the reference interval), or borderline (≤3 mmol/L higher than the high end of the reference interval) corrected hyper[Cl^−^].

During the 60‐month study period, raw data including 62,226 samples from dogs and 14,972 samples from cats were extracted from the electronic medical records. A total of 17,120 dogs and 4197 cats were ultimately included in the analysis, each with at least one measurement of blood [Cl^−^] and [Na^+^]. Of these animals, 58.7% (10,047/17,120) dogs and 31.6% (1326/4197) cats had normal measured [Cl^−^]. In animals with normal measured [Cl^−^], the case‐fatality rate was 4.9% (493/10,047) in dogs (Figure 1A) and 5.0% (66/1326) in cats (Figure 1B). Regarding the corrected [Cl^−^], 65.0% (11,125/17,120) dogs and 56.0% (2350/4197) cats exhibited normal corrected [Cl^−^]. Of dogs with normal corrected [Cl^−^], the case‐fatality rate was 4.8% (535/11,125) (Figure 2A), and the case‐fatality rate was 4.6% (109/2350) in cats (Figure 2B).

### 3.1 Measured Hyper[Cl^−^]

Measured hyper[Cl^−^] was diagnosed in 18.1% (3092/17,120) dogs and 9.4% (396/4197) cats. Of these animals, 68.2% (2110/3092) dogs and 62.9% (249/396) cats exhibited borderline measured hyper[Cl^−^], while 18.0% (557/3092) dogs and 22.0% (87/396) cats showed mild measured hyper[Cl^−^]. Moderate measured hyper[Cl^−^] was observed in 8.3% (258/3092) dogs and 8.8% (35/396) cats, whereas severe hyper[Cl^−^]_measured_ was present in 5.4% (167/3092) of dogs and 6.3% (25/396) of cats (Table 1).

**TABLE 1:** Prevalence of normal chloride concentration and hyperchloremia in dogs and cats presented to a tertiary referral veterinary hospital over a 60‐month period.

	Dogs		Cats	
	Proportion (%)	Number (*n*)	Proportion (%)	Number (*n*)
All measured and corrected chloride		17,120		4197
Normal measured chloride	58.7	10,047/17,120	31.6	1326/4197
Normal corrected chloride	65.0	11,125/17,120	56.0	2350/4197
All measured hyperchloremia	18.1	3092/17,120	9.4	396/4197
Borderline	68.2	2110/3092	62.9	249/396
Mild	18.0	557/3092	22.0	87/396
Moderate	8.3	258/3092	8.8	35/396
Severe	5.4	167/3092	6.3	25/396
All corrected hyperchloremia	21.1	3607/17,120	9.1	384/4197
Borderline	77.6	2798/3607	72.4	278/384
Mild	16.0	577/3607	19.5	75/384
Moderate	4.1	149/3607	4.4	17/384
Severe	2.3	83/3607	3.6	14/384

*Note*: Animals with blood or serum corrected [Cl^−^] higher than the reference intervals were identified and categorized as severe (≥10 mmol/L higher than the high end of the reference interval), moderate (7–9 mmol/L higher than the high end of the reference interval), mild (4–6 mmol/L higher than the high end of the reference interval), or borderline (≤3 mmol/L higher than the high end of the reference interval) corrected hyper[Cl^−^].

The overall case‐fatality rates in dogs and cats with measured hyper[Cl^−^] were 15.7% (484/3092) and 21.0% (83/396), respectively. Specifically, in dogs, the case‐fatality rates were 10.8% (228/2110) for those with borderline measured hyper[Cl^−^], 15.8% (88/557) for mild measured hyper[Cl^−^], 38.4% (99/258) for moderate measured hyper[Cl^−^], and 41.3% (69/167) for severe measured hyper[Cl^−^] (Figure 1A). In cats, the case‐fatality rates were 18.1% (45/249) for borderline measured hyper[Cl^−^], 20.7% (18/87) for mild measured hyper[Cl^−^], 31.4% (11/35) for moderate measured hyper[Cl^−^], and 36.0% (9/25) for severe measured hyper[Cl^−^] (Figure 1B).

The odds ratio (OR) for nonsurvival when combining all degrees of measured hyper[Cl^−^] was 3.60 (95% confidence interval [CI] 3.15–4.11; *p* < 0.0001) in dogs and 5.45 (95% CI: 4.01–7.42; *p* < 0.0001) in cats. The severity of measured hyper[Cl^−^] showed a linear association with higher case‐fatality rates in dogs and cats (*p* < 0.0001). Post hoc analysis revealed that borderline (OR: 2.35 [95% CI: 1.99–2.77]; *p* < 0.0001), mild (OR: 3.65 [95% CI: 2.86–4.66]; *p* < 0.0001), moderate (OR: 12.08 [95% CI: 9.26–15.76]; *p* < 0.0001), and severe corrected hyper[Cl^−^] (OR: 13.66 [95% CI: 9.92–18.81]; *p* < 0.0001) were associated with a higher fatality rate compared with normal measured [Cl^−^] in dogs. In cats, borderline (OR: 4.56 [95% CI: 3.13–6.62]; *p* < 0.0001), mild (OR: 5.45 [95% CI: 3.15–9.42]; *p* < 0.0001), moderate (OR: 9.61 [95% CI: 4.65–19.87]; *p* < 0.0001), and severe measured hyper[Cl^−^] (OR: 11.79 [95% CI: 5.19–26.79]; *p* < 0.0001) were associated with a higher case‐fatality rate compared with normal measured [Cl^−^].

### 3.2 Corrected Hyper[Cl^−^]

A total of 3607 of 17,120 dogs (21.1%) and 384 of 4197 cats (9.1%) were diagnosed with corrected hyper[Cl^−^]. Among these animals, 77.6% (2798/3607) dogs and 72.4% (278/384) cats exhibited borderline corrected hyper[Cl^−^], while 16.0% (577/3607) dogs and 19.5% (75/384) cats had mild corrected hyper[Cl^−^]. Additionally, 4.1% (149/3607) dogs and 4.4% (17/384) cats had moderate corrected hyper[Cl^−^], while 2.3% (83/3607) dogs and 3.6% (14/384) cats had severe corrected hyper[Cl^−^] (Table 1).

The overall case‐fatality rate for animals with corrected hyper[Cl^−^] was 13.5% (486/3607) in dogs and 24.5% (94/384) in cats. Among dogs, the case‐fatality rates for borderline, mild, moderate, and severe hyper[Cl^−^] were 11.5% (323/2798), 18.7% (108/577), 26.2% (39/149), and 19.3% (16/83), respectively (Figure 2A). In cats, the case‐fatality rates for borderline, mild, moderate, and severe hyper[Cl^−^] were 25.5% (71/278), 22.7% (17/75), 23.5% (4/17), and 14.3% (2/14), respectively (Figure 2B).

There was an association between corrected hyper[Cl^−^] and higher case‐fatality rates in both dogs and cats (*p* < 0.0001). The OR for nonsurvival with any degree of corrected hyper[Cl^−^] was 3.1 (95% CI: 2.76–3.58; *p* < 0.0001) in dogs and 6.66 (95% CI: 4.93–9.0) in cats. The severity of corrected hyper[Cl^−^] showed a linear association with higher case‐fatality rates in dogs (*p* < 0.0001) but not in cats (*p* = 0.34). Post hoc analysis revealed that borderline (OR: 2.64 [95% CI: 2.28–3.05]; *p* < 0.0001), mild (OR: 4.66 [95% CI: 3.72–5.84]; *p* < 0.0001), moderate (OR: 7.21 [95% CI: 4.96–10.47]; *p* < 0.0001), and severe corrected hyper[Cl^−^] (OR: 4.93 [95% CI: 2.86–8.5]; *p* < 0.0001) were associated with a higher case‐fatality rate compared with normal corrected [Cl^−^] in dogs. In cats, borderline (OR: 7.05 [95% CI: 5.07–9.81]; *p* < 0.0001), mild (OR: 6.12 [95% CI: 3.47–10.8]; *p* < 0.0001), and moderate corrected hyper[Cl^−^] (OR: 6.82 [95% CI: 2.31–20.2]; *p* = 0.0003) were associated with a higher case‐fatality rate compared with normal corrected [Cl^−^]. However, severe corrected hyper[Cl^−^] was not associated with a higher case‐fatality rate compared with normal corrected [Cl^−^] in cats (*p* = 0.14).

### 3.3 Measured Versus Corrected Hyper[Cl^−^]

The fatality rates of dogs with measured and corrected hyper[Cl^−^] were compared. In dogs, the overall fatality rate was higher in dogs with measured hyper[Cl^−^] than in dogs with corrected hyper[Cl^−^] (*p* = 0.01). There was a difference in fatality rates for moderate (*p* = 0.01) and severe hyper[Cl^−^] (*p* = 0.0006), while no differences were found in fatality rates for borderline (*p* = 0.42) and mild hyper[Cl^−^] (*p* = 0.19) when comparing the two types.

In cats, the overall fatality rate was not different between measured and corrected hyper[Cl^−^] (*p* = 0.24). The case‐fatality rates for cats with borderline (*p* = 0.039) was higher with corrected hyper[Cl^−^] than those with measured hyper[Cl^−^], but mild (*p* = 0.76), moderate (*p* = 0.55), and severe hyper[Cl^−^] (*p* = 0.27) were not higher with corrected hyper[Cl^−^] than with measured hyper[Cl^−^].

### 3.4 Prehospital Versus Hospital‐Acquired Corrected Hyper[Cl^−^]

Among animals diagnosed with corrected hyper[Cl^−^], 50.9% (1835/3607) of dogs and 38.3% (147/384) of cats were classified as having prehospital corrected hyper[Cl^−^], while 39.5% (1424/3607) of dogs and 48.7% (187/384) of cats were categorized as hospital‐acquired corrected hyper[Cl^−^] (Tables 2 and 3). A total of 9.6% (348/3607) dogs and 13.0% (50/384) cats with corrected hyper[Cl^−^] at presentation had received medical or surgical treatment from another veterinarian before being referred to our hospital, and in such cases, the time of onset of corrected hyper[Cl^−^] was reported as unknown. The case‐fatality rate for hospital‐acquired corrected hyper[Cl^−^] was higher than prehospital corrected hyper[Cl^−^] in dogs (OR: 1.70 [95% CI: 0.7–2.08]; *p* < 0.0001), but there was no difference in cats (OR: 0.97 [95% CI: 0.6–1.59]; *p* = 0.9). Notably, in dogs, there was a difference in the fatality rate among those that had prehospital versus hospital‐acquired, borderline corrected hyper[Cl^−^] (OR: 2.04 [95% CI: 1.6–2.93]; *p* < 0.0001).

**TABLE 2:** Prehospital versus hospital‐acquired corrected hyperchloremia in 3607 dogs with corrected hyperchloremia.

	Prehospital (*n* = 1835)			Hospital‐acquired (*n* = 1424)		
	Nonsurvivors	Survivors	Fatality	Nonsurvivors	Survivors	Fatality				
	(*n*)	(*n*)	(%)	(*n*)	(*n*)	(%)	*p*‐value	OR	95% CI	
Borderline (*n* = 2798)	121	1294	8.6	183	959	16.0	<0.0001	2.04	1.60	2.93
Mild (*n* = 577)	52	248	17.3	47	150	23.9	0.085	1.49	0.96	2.31
Moderate (*n* = 149)	23	52	30.7	13	41	24.1	0.14	0.49	0.20	1.17
Severe (*n* = 83)	8	37	17.8	7	24	22.6	0.77	1.35	0.46	3.88
Total (*n* = 3607)	204	1631	11.1	250	1174	17.6	<0.0001	1.70	0.70	2.08

*Note*: Of these 3607 dogs, 1835 were categorized as having prehospital corrected hyperchloremia, while 1424 were categorized as having hospital‐acquired corrected hyperchloremia. Cases with an unknown outcome (348 dogs) are included in the total. Dogs with blood or serum [Cl^−^]_corrected_ higher than the reference intervals were identified and categorized as severe (≥10 mmol/L higher than the high end of the reference interval), moderate (7–9 mmol/L higher than the high end of the reference interval), mild (4–6 mmol/L higher than the high end of the reference interval), or borderline (≤3 mmol/L higher than the high end of the reference interval) corrected hyper[Cl^−^]. The *p*‐values were obtained by comparing the fatality rates of prehospital corrected hyper[Cl^−^] and hospital‐acquired corrected hyper[Cl^−^] in different severities of corrected hyper[Cl^−^] in dogs.

Abbreviations: CI, confidence interval; OR, odds ratio.

**TABLE 3:** Prehospital versus hospital‐acquired corrected hyperchloremia in 384 cats with corrected hyperchloremia.

	Prehospital (*n* = 147)			Hospital‐acquired (*n* = 187)		
	Nonsurvivors	Survivors	Fatality	Nonsurvivors	Survivors	Fatality				
	(*n*)	(*n*)	(%)	(*n*)	(*n*)	(%)	*p*‐value	OR	95% CI	
Borderline (*n* = 278)	27	76	26.2	41	100	29.1	0.67	1.15	0.65	2.05
Mild (*n* = 75)	10	19	34.5	7	27	20.6	0.26	0.49	0.17	1.55
Moderate (*n* = 17)	4	4	50.0	1	6	14.3	0.28	0.17	0.012	2.20
Severe (*n* = 14)	0	7	0	2	3	40.0	0.15	—	0.72	—
Total (*n* = 384)	41	106	27.9	51	136	27.3	0.90	0.97	0.60	1.59

*Note*: Of these 384 cats, 147 cats were categorized as having prehospital corrected hyperchloremia, while 187 cats were categorized as having hospital‐acquired corrected hyperchloremia. Cases with an unknown outcome (50 cats) are included in the total. Cats with blood or serum corrected [Cl^−^] higher than the reference intervals were identified and categorized as severe (≥10 mmol/L higher than the high end of the reference interval), moderate (7–9 mmol/L higher than the high end of the reference interval), mild (4–6 mmol/L higher than the high end of the reference interval), or borderline (≤3 mmol/L higher than the high end of the reference interval) corrected hyper[Cl^−^]. The *p*‐values were obtained by comparing the fatality rates of prehospital corrected hyper[Cl^−^] and hospital‐acquired corrected hyper[Cl^−^] in different severities of corrected hyper[Cl^−^] in cats.

Abbreviations: CI, confidence interval; OR, odds ratio.

### 3.5 The Primary Disease Processes Associated With Corrected Hyper[Cl^−^]

The most common primary disease processes observed in dogs with corrected hyper[Cl^−^] were neurologic (241/809 [29.8%]), neoplastic (182 [22.5%]), and gastrointestinal disease (181 [22.4%]) (Table 4). Among cats with corrected hyper[Cl^−^], the most prevalent primary disease processes reported were urologic (36/106 [34%]), cardiovascular (26 [24.5%]), and gastrointestinal disease (25 [23.6%]) (Table 5). Multiple comorbidities were found in both dogs and cats with corrected hyper[Cl^−^]. The proportion of animals with each primary disease process was also investigated. In dogs, corrected hyper[Cl^−^] was noted in 31.3% (45/144) dogs with sepsis, 29.4% (10/34) with CPA, and 22.2% (50/225) with pancreatic disease (Table 6). In cats, corrected hyper[Cl^−^] was noted in 50% (4/8) cats with CPA, 24% (12/50) with sepsis, and 11.8% (4/34) with DM or DKA (Table 7). When Bonferroni correction was applied, various primary disease processes were associated with the development of measured hyper[Cl^−^]. In dogs, these primary disease processes included sepsis, CPA, and pancreatic, respiratory, cardiovascular, hematologic, and gastrointestinal diseases. In cats, CPA and sepsis were the primary disease processes.

**TABLE 4:** Primary disease processes of 809 dogs with mild (577 dogs), moderate (149 dogs), or severe (83 dogs) corrected hyperchloremia.

	Severe (*n*)	Moderate (*n*)	Mild (*n*)	Total (*n*)	%
Neurologic	32	56	153	241	29.8
Neoplastic	20	26	136	182	22.5
Gastrointestinal	22	34	125	181	22.4
Urologic	13	33	117	163	20.1
Respiratory	19	30	114	163	20.1
Cardiovascular	9	16	96	121	15.0
Hepatobiliary	11	19	88	118	14.6
Musculoskeletal	5	18	59	82	10.1
Hematologic	5	17	59	81	10.0
Pancreatic	5	12	33	50	6.2
Oropharyngeal	3	12	31	46	5.7
Sepsis	5	8	32	45	5.6
Toxicoses	3	2	24	29	3.6
DM/DKA	4	3	13	20	2.5
Reproductive	3	2	13	18	2.2
Hypothyroidism	2	3	8	13	1.6
CPA	0	3	7	10	1.2
Hypoadrenocorticism	4	0	5	9	1.1
Hyperadrenocorticism	3	2	4	9	1.1
Hyperthyroidism	0	0	1	1	0.1
Diabetes insipidus	0	1	0	1	0.1

*Note*: Individual animals may have more than one condition. Dogs with blood or serum [Cl^−^]_corrected_ higher than the reference intervals were identified and categorized as severe (≥10 mmol/L higher than the high end of the reference interval), moderate (7–9 mmol/L higher than the high end of the reference interval), or mild (4–6 mmol/L higher than the high end of the reference interval) corrected hyper[Cl^−^].

Abbreviations: [Cl^−^]_corrected_, corrected blood chloride concentration; CPA, cardiopulmonary arrest; DM/DKA, diabetes mellitus/diabetic ketoacidosis.

**TABLE 5:** Primary disease processes of 106 cats with mild (75 cats), moderate (17 cats), or severe (14 cats) corrected hyperchloremia.

	Severe (*n*)	Moderate (*n*)	Mild (*n*)	Total (*n*)	%
Urologic	4	4	28	36	34.0
Cardiovascular	5	2	19	26	24.5
Gastrointestinal	5	1	19	25	23.6
Neoplastic	4	2	18	24	22.6
Respiratory	2	3	7	12	11.3
Hepatobiliary	2	0	10	12	11.3
Neurologic	1	4	7	12	11.3
Hematologic	1	1	10	12	11.3
Septic	1	2	9	12	11.3
Musculoskeletal	1	2	9	12	11.3
Hyperthyroidism	1	1	6	8	7.5
Oropharyngeal	0	2	4	6	5.7
DM/DKA	0	0	4	4	3.8
Pancreatic	0	1	3	4	3.8
CPA	1	1	2	4	3.8
Hyperadrenocorticism	0	0	1	1	0.9
Reproductive	0	0	1	1	0.9
Toxicoses	0	0	1	1	0.9
Hypoadrenocorticism	0	0	0	0	0
Hypothyroidism	0	0	0	0	0
Diabetes insipidus	0	0	0	0	0

*Note*: Individual animals may have more than one condition. Cats with blood or serum [Cl^−^]_corrected_ higher than the reference intervals were identified and categorized as severe (≥10 mmol/L higher than the high end of the reference interval), moderate (7–9 mmol/L higher than the high end of the reference interval), or mild (4–6 mmol/L higher than the high end of the reference interval) corrected hyper[Cl^−^].

Abbreviations: [Cl^−^]_corrected_, corrected blood chloride concentration; CPA, cardiopulmonary arrest; DM/DKA, diabetes mellitus/diabetic ketoacidosis.

**TABLE 6:** The proportion of dogs with mild (577 dogs), moderate (149 dogs), and severe (83 dogs) corrected hyperchloremia versus normal corrected chloride (11,125 dogs) with each primary disease process in a total of 11,934 dogs.

Disease process	Normal corrected [Cl^−^] (n)	Corrected hyper[Cl^−^] (*n*)	Total (*n*)	Proportions (%)	OR	*p*‐value	95% CI	
Sepsis	99	45	144	31.3	4.47	<0.0001	3.13	6.38
CPA	24	10	34	29.4	4.02	0.0009	1.97	8.11
Pancreatic	175	50	225	22.2	2.80	<0.001	2.03	3.84
Respiratory	814	163	977	16.7	2.03	<0.0001	1.70	2.42
Cardiovascular	619	121	740	16.4	1.95	<0.0001	1.42	2.38
DM/DKA	101	20	121	16.5	1.91	0.012	1.18	3.10
Hematologic	424	81	505	16.0	1.88	<0.0001	1.48	2.40
Toxicoses	179	29	208	13.9	1.57	0.031	0.94	2.32
Gastrointestinal	1212	181	1393	13.0	1.49	<0.0001	1.26	3.52
Hepatobiliary	823	118	941	12.5	1.41	0.0013	1.02	1.74
Hypoadrenocorticism	68	9	77	11.7	1.27	0.44	0.66	2.52
Reproductive	171	18	189	9.5	1.01	0.90	0.55	1.64
Neurology	2574	241	2815	8.6	0.88	0.090	0.76	2.04
Diabetes insipidus	11	1	12	8.3	0.87	>0.99	0.09	6.12
Urologic	1839	163	2002	8.1	0.83	0.038	0.70	0.99
Hypothyroidism	214	13	227	5.7	0.58	0.052	0.33	1.00
Neoplasia	2874	182	3056	6.0	0.56	<0.0001	0.42	1.31
Musculoskeletal	1349	82	1431	5.7	0.56	<0.0001	0.40	0.71
Hyperadrenocorticism	159	9	168	5.4	0.54	0.083	0.29	1.04
Oropharyngeal	1470	46	1516	3.0	0.28	<0.0001	0.18	0.37

*Note*: Individual animals may have more than one condition. The *p*‐values were obtained by comparing the proportion of normal [Cl^−^]_corrected_ and corrected hyper[Cl^−^] among the total number of dogs with each primary disease process. After Bonferroni correction, *p* < 0.002 was considered statistically significant.

Abbreviations: [Cl^−^]_corrected_, corrected blood chloride concentration; CI, confidence interval; CPA, cardiopulmonary arrest; DM/DKA, diabetes mellitus/diabetic ketoacidosis; OR, odds ratio.

**TABLE 7:** The proportion of cats with mild (75 cats), moderate (17 cats), and severe (14 cats) corrected hyperchloremia versus normal corrected chloride (1326 cats) with each primary disease process in a total of 1432 cats.

Primary diseases	Normal corrected [Cl^−^] (*n*)	Corrected hyper[Cl^−^] (*n*)	Total (*n*)	Proportion (%)	OR	*p*‐value	95% CI	
CPA	4	4	8	50.0	16.9	0.0006	4.86	58.1
Sepsis	38	12	50	24.0	5.54	<0.0001	2.84	10.5
DM/DKA	30	4	34	11.8	2.23	0.13	0.84	6.11
Reproductive	9	1	10	10.0	1.84	0.44	0.17	11.1
Cardiovascular	278	26	304	8.6	1.63	0.037	1.06	2.47
Hepatobiliary	135	12	147	8.2	1.50	0.2	0.80	2.75
Hematologic	148	12	160	7.5	1.36	0.3	0.73	2.49
Musculoskeletal	156	12	168	7.1	1.29	0.39	0.69	2.35
Neurology	179	12	191	6.3	1.12	0.75	0.60	2.02
Gastrointestinal	376	25	401	6.2	1.11	0.65	0.73	1.69
Urologic	575	36	611	5.9	1.04	0.85	0.72	1.51
Respiratory	200	12	212	5.7	0.99	>0.99	0.54	1.79
Hyperthyroidism	166	8	174	4.6	0.79	0.62	0.38	1.59
Pancreatic	84	4	88	4.6	0.78	0.82	0.30	2.03
Neoplasia	599	24	623	3.9	0.62	0.029	0.40	0.94
Toxicoses	35	1	36	2.8	0.47	0.72	0.05	2.64
Oropharyngeal	473	6	479	1.3	0.19	<0.0001	0.09	0.40

*Note*: Individual animals may have more than one condition. The *p*‐values were obtained by comparing the proportion of normal [Cl^−^]_corrected_ and corrected hyper[Cl^−^] among the total number of cats with each primary disease process. After Bonferroni correction, *p* < 0.002 was considered statistically significant.

Abbreviations: [Cl^−^]_corrected_, corrected blood chloride concentration; CI, confidence interval; CPA, cardiopulmonary arrest; DM/DKA, diabetes mellitus/diabetic ketoacidosis; OR, odds ratio.

### 3.6 Absolute Difference of Corrected [Cl^−^] During Hospitalization

**FIGURE 3:**
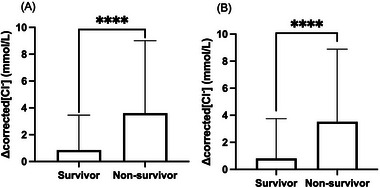
(A) The absolute difference between the maximal and minimal corrected chloride concentration (∆[Cl^−^]_corrected_) during hospitalization was compared between the survivors and nonsurvivors in dogs. Dogs that died or were euthanized during hospitalization had significantly higher ∆[Cl^−^]_corrected_ that those that survived (*p* < 0.0001). Boxes represent the interquartile ranges (25th to 75th percentiles), while whiskers represent 1.5 times the interquartile ranges. (B) The absolute difference between the maximal and minimal corrected chloride concentration (∆[Cl^−^]_corrected_) during hospitalization was compared between the survivors and nonsurvivors in cats. Cats that died or were euthanized during hospitalization had significantly higher ∆[Cl^−^]_corrected_ that those that survived (*p* < 0.0001). Boxes represent the interquartile ranges (25th to 75th percentiles), while whiskers represent 1.5 times the interquartile ranges.

A total of 14,973 visits in dogs and 2417 visits in cats had more than one corrected [Cl^−^] measurement during each hospitalization. In dogs, 96% (9625/10,026) with multiple corrected [Cl^−^] measurements survived. In cats, 94.7% (2288/2417) with multiple corrected [Cl^−^] measurements survived. In dogs, the ∆[Cl^−^]_corrected_ was 0.8 ± 2.6 mmol/L in the survivor group and 3.6 ± 5.4 mmol/L in the nonsurvivor group (Figure 3A). In cats, the ∆[Cl^−^]_corrected_ was 0.8 ± 2.9 mmol/L in the survivor group and 3.5 ± 5.4 mmol/L in the nonsurvivor group (Figure 3B). Both dogs and cats that died or were euthanized during hospitalization exhibited higher ∆[Cl^−^]_corrected_ values compared with those that survived (*p* < 0.0001).

## 4 Discussion

Increased measured and corrected hyper[Cl^−^] were observed commonly in dogs and cats at our hospital. Notably, cases with corrected hyper[Cl^−^] exhibited higher fatality rates compared with those with normal corrected [Cl^−^]. This association remained significant even in dogs and cats with borderline corrected hyper[Cl^−^]. Additionally, the severity of corrected hyper[Cl^−^] was directly proportional to the increase in case‐fatality rate in dogs, though this was not the case in cats. The linear relationship in dogs aligns with similar findings reported in human and veterinary patients [16, 17] and can be attributed to several mechanisms. Several studies conducted in human patients have demonstrated that corrected hyper[Cl^−^] is linked to a higher likelihood of developing AKI, which consequently leads to worse outcomes [2, 15, 27, 28, 29, 30]. The presence of severe corrected hyper[Cl^−^], particularly when accompanied by metabolic acidosis, contributes to coagulopathy, proinflammation, and immune dysfunction [3, 31, 32, 33, 34, 35]. However, the exact mechanisms responsible for these effects have not been fully elucidated [36]. Indeed, it is also plausible that corrected hyper[Cl^−^] merely acts as an indicator of the severity of the underlying disease rather than being a direct cause of poor outcomes.

It was observed that corrected hyper[Cl^−^] frequently developed during hospitalization in dogs (39.5%) and cats (48.7%). These findings align with similar reports in human patients and suggest that the development of corrected hyper[Cl^−^] may often be related to therapeutic interventions or the progression of underlying diseases during hospitalization [2, 37]. Furthermore, the case‐fatality rates of dogs with hospital‐acquired corrected hyper[Cl^−^] were significantly higher than those with corrected hyper[Cl^−^] present at presentation. In human medicine, there has been growing interest in how the outcomes in critically ill patients are influenced by iatrogenic corrected hyper[Cl^−^] [15, 36, 38, 39]. One potential cause of hospital‐acquired corrected hyper[Cl^−^] is the administration of chloride‐rich fluids, including 0.9% sodium chloride [14, 15, 22, 27, 38, 40, 41, 42, 43, 44, 45, 46]. Several large randomized controlled studies in human patients comparing the outcomes of intravenous administration of 0.9% sodium chloride versus balanced electrolyte crystalloid solutions have reported conflicting results [22, 43, 44, 46]. Previous studies found an association between unfavorable outcomes and the administration of 0.9% sodium chloride, although the latest randomized controlled trial and meta‐analysis did not report this adverse effect [47]. In veterinary medicine, a recent prospective study in dogs found no association between mortality and chloride load, but an association was observed between maximal corrected [Cl^−^] during hospitalization and the development of AKI [23]. Although our study did not investigate the specific causes of hospital‐acquired corrected hyper[Cl^−^] and its association with higher case‐fatality rates, it is possible that the development of corrected hyper[Cl^−^] during hospitalization could be related to the therapeutic interventions or the progression of the primary diseases during the hospital stay. In addition, the post hoc analysis revealed that only borderline corrected hyper[Cl^−^] was associated with a higher case‐fatality rate in dogs with hospital‐acquired corrected hyper[Cl^−^] compared with those with prehospital corrected hyper[Cl^−^]. This finding suggests that even a slight increase in corrected hyper[Cl^−^] during hospitalization may be associated with poor outcomes. It may be that the mechanisms leading to borderline hyper[Cl^−^] differ from those associated with mild, moderate, and severe hyper[Cl^−^], possibly due to different medical interventions or underlying disease conditions; however, this finding may reflect the relative ease in finding this association given the large number of dogs with borderline corrected hyper[Cl^−^]. In cats, no difference in the case‐fatality rate was observed with prehospital versus hospital‐acquired corrected hyper[Cl^−^]. This lack of difference may be attributed to the small sample size of cats with corrected hyper[Cl^−^]. Alternately, the etiology and mechanisms underlying corrected hyper[Cl^−^] in cats may differ from those in dogs and people.

The current study revealed an association between fluctuations in corrected [Cl^−^] during hospitalization and nonsurvival. Similar associations have been reported in several retrospective and prospective studies involving human patients [28, 48, 49]. One study found that an increase in ∆[Cl^−^] >5 mmol/L within 72 h after admission, even without the development of hypernatremia, was associated with the development of AKI [28]. Interestingly, it also showed that both positive and negative fluctuations in [Cl^−^] after admission to the ICU were linked to AKI development [28]. In the current study, only the absolute difference between the maximal and minimal corrected [Cl^−^] was evaluated, and thus, the associations with specific positive and negative fluctuations in [Cl^−^] could not be assessed. A recent retrospective feline study reported that nonsurviving cats exhibited a higher degree of ∆[Cl^−^] compared with surviving cats [16]. However, once euthanized cases were excluded, ∆[Cl^−^] in cats that died was not different from that in survivors. In the current study, the nonsurvivor group included both euthanized cases and those that died naturally. How ∆[Cl^−^] may have influenced euthanasia was not investigated. Further studies are necessary to determine the precise relationship between the fluctuation of corrected [Cl^−^], nonsurvival, and development of AKI and to assess whether the prognosis of dogs and cats could be improved by preventing fluctuations in chloride concentration.

In the studied population, neurologic diseases were the most commonly reported primary disease processes in dogs with corrected hyper[Cl^−^]. In human patients, several neurologic diseases, such as acute ischemic stroke, intracerebral or subarachnoid hemorrhages, and traumatic brain injury, have been associated with the development of hyper[Cl^−^] [50, 51, 52, 53]. One study demonstrated a significant correlation between the severity of neurologic diseases and the occurrence of hyper[Cl^−^] [51]. Previous studies have found that human patients with neurologic diseases who developed hyper[Cl^−^] had a higher incidence of AKI, which in turn could contribute to a higher mortality rate in the current patient cohort [49, 50, 53].

Urologic diseases were the most common primary disease process found in cats with corrected hyper[Cl^−^]. Similar to the previous discussion, the association between hyper[Cl^−^] and the development of AKI as a consequence of therapeutic intervention has been extensively investigated in human patients [21, 22, 54]. Moreover, several studies in human patients have indicated that hyper[Cl^−^] is a prevalent electrolyte abnormality in individuals with end‐stage kidney disease, and the progression of chronic kidney disease is correlated with the worsening of hyper[Cl^−^] [55, 56, 57]. These findings suggest that kidney disease may also contribute to the development of corrected hyper[Cl^−^] in both dogs and cats.

Although sepsis was not the most common primary disease process observed in dogs and cats with corrected hyper[Cl^−^] in the current study, it is worth noting that a substantial proportion of dogs and cats with sepsis did exhibit corrected hyper[Cl^−^]. Several studies in human patients with sepsis or systemic inflammatory response syndrome have reported a correlation between hyper[Cl^−^] and hospital mortality [28, 29, 30, 58]. This association can be attributed to factors such as reduced renal blood flow resulting in the development of AKI, coagulopathy, augmented proinflammatory response, and altered neutrophil function due to hyper[Cl^−^] and metabolic acidosis [3, 31, 33, 59, 60, 61, 62, 63]. Further studies are warranted to determine the impact of corrected hyper[Cl^−^] on morbidity and mortality in dogs and cats with sepsis.

This study has several inherent limitations given its retrospective design. Although the tertiary referral hospital where the samples were collected and processed has standard protocols to maintain quality, we cannot guarantee these protocols were followed in every case due to the retrospective design of the study. Because records often do not state a clear reason for euthanasia or a specific cause of death, natural death and euthanasia were grouped together in the nonsurvivor group. Excluding cases of euthanasia might have changed the observed association between hyper[Cl^−^] and case‐fatality rate. Due to limited data regarding the timing of blood sampling in relation to therapeutic interventions, it is challenging to ascertain the precise effects of medical interventions on chloride and sodium concentrations. Additionally, acid–base status was not reported for the animals included in this study, as the acid–base data were unavailable from the serum biochemistry profiles. In previous studies, 66% of dogs and 41% of cats with metabolic acidosis were also diagnosed with corrected hyper[Cl^−^] [10, 64]. Thus, it is important to evaluate the impact of corrected hyper[Cl^−^] in dogs and cats in the context of their acid–base status. Furthermore, the primary disease processes associated with corrected [Cl^−^] were based on the final diagnoses determined by the primary clinicians. These underlying diseases are likely related to corrected hyper[Cl^−^]; however, it does not necessarily imply that they are the primary pathophysiological mechanisms leading to corrected hyper[Cl^−^]. Last, the current study was unable to establish the true prevalence of corrected hyper[Cl^−^] in dogs and cats because only animals with blood or serum chloride concentrations measured at a single tertiary referral hospital were included. Further multicenter studies are needed to determine the actual prevalence of abnormal [Cl^−^]_corrected_, its association with underlying diseases, and its influence on mortality.

In conclusion, results of this cohort study found that corrected hyper[Cl^−^] was common. The overall case‐fatality rates of animals with corrected hyper[Cl^−^] were higher than those of animals with normal corrected [Cl^−^]. In addition, there was a linear association between a higher case‐fatality rate and the severity of corrected hyper[Cl^−^] in dogs. Notably, the case‐fatality rate was higher in dogs with hospital‐acquired corrected hyper[Cl^−^] than with prehospital corrected hyper[Cl^−^]. Future prospective studies are warranted to determine if abnormal corrected [Cl^−^] is important as a direct cause of morbidity or a marker of disease severity.

## Author Contributions

**Yu Ueda**: conceptualization, data curation, formal analysis, investigation, methodology, project administration, software, visualization, writing – original draft. **Steven E. Epstein**: conceptualization, formal analysis, investigation, methodology, project administration, resources, software, supervision, validation, writing – review and editing. **Kate Hopper**: conceptualization, investigation, methodology, project administration, resources, software, supervision, writing – review and editing.

## Conflicts of Interest

Dr. Epstein is an Assistant Editor of the Journal but only participated in the peer review process as an author. The authors declare no other conflicts of interest.

## Endnotes

1 ABL 705, Radiometer Medical A/S, Copenhagen, Denmark.

2 Chemistry Analyzer, Hitachi 917 and Hitachi c501, Roche Diagnostics, Indianapolis, IN.

3 GraphPad Software Prism Version 9.3.1, San Diego, CA.
